# The scanning electron microscope in microbiology and diagnosis of infectious disease

**DOI:** 10.1038/srep26516

**Published:** 2016-05-23

**Authors:** Christine G. Golding, Lindsey L. Lamboo, Daniel R. Beniac, Timothy F. Booth

**Affiliations:** 1 Viral Diseases Division, National Microbiology Laboratory, Public Health Agency of Canada, Winnipeg, Manitoba, Canada; 2 Department of Medical Microbiology, University of Manitoba, Winnipeg, Manitoba, Canada

## Abstract

Despite being an excellent tool for investigating ultrastructure, scanning electron microscopy (SEM) is less frequently used than transmission electron microscopy for microbes such as viruses or bacteria. Here we describe rapid methods that allow SEM imaging of fully hydrated, unfixed microbes without using conventional sample preparation methods. We demonstrate improved ultrastructural preservation, with greatly reduced dehydration and shrinkage, for specimens including bacteria and viruses such as Ebola virus using infiltration with ionic liquid on conducting filter substrates for SEM.

In early studies, electron microscopy was pivotal in helping to identify the causative agents of infectious diseases^1^. It is still an important technique that can help to diagnose pathogens, and in testing to identify microorganisms^2^. Traditionally, negative staining for transmission electron microscopy (TEM) has been the “gold standard” for imaging microbial samples, for example in diagnostic virology^3^. However, negative-stain TEM requires an adequate concentration of bacterial cells or virus particles, since these are adsorbed to a thin support film. Thus microbes need to be grown to a high tire and/or concentrated by centrifugation, which is often not possible with patient specimens or agents that are not culturable. As a result, electron microscopy has historically suffered from low test sensitivity for many types of microbiological investigations. The detection of agents such as poxviruses or polyoma viruses in patient specimens usually requires a minimum concentration of between 10^5^ to 10^6^ particles/ml for TEM^4^,^5^. By comparison, the level of detection of viruses using culture or nucleic acid testing usually ranges between 1 and 50 particles per assay^6^. The recent development of filtration techniques show that both TEM and SEM identification of viruses can be carried out with as little as 5000 total particles per sample^7^. Moreover, electron microscopy is useful for identifying the type of microbe present, often to genus, allowing the selection of more specific tests (for example primers or specific antibodies) to fully identify the agents present. Electron microscopy is thus an ideal “catch all” method giving an “open view” for situations where a novel or emerging pathogen is being investigated where there is no *a priori* knowledge of the type of agent present^6^.

The scanning electron microscope (SEM) can also be useful to reveal morphological features of isolated organisms as well as for diagnosis, but difficulty with specimen preparation methods have in the past limited the use of SEM for routine microbiology^7^,^8^. Nowadays extremely high quality polycarbonate filters are available: the optimum pore size can be selected to collect any virus or bacterial species (the pores can be as small as 10 nm, less than the smallest viruses). These filters are suitable for surface observation of viruses and bacteria by SEM^7^. Two main problems occur with obtaining high resolution SEM images of microbes. Firstly, in order to get adequate contrast, and to reduce charging for small organic particles such as bacteria and viruses at magnifications greater than 1000 x, a conducting surface is needed. Secondly, biological specimens have traditionally needed to be dehydrated for the best imaging performance in the SEM. If a wet specimen is placed in the microscope, operation under high vacuum conditions tends to dry the specimen out quickly. Both of these factors compromise microscope performance and can reduce contrast and resolution. During SEM observation drying is a problem, and usually causes collapse, shrinkage, and distortion of the specimen, even after preservation by chemical fixation. Previously, a variety of methods have been developed to dehydrate specimens prior to SEM observation, using solvents, sometimes in conjunction with critical point drying, or by freeze drying. Alternatively, methods to image specimens in the hydrated state have been used, employing “wet-SEM”^7^,^9^,^10^,^11^, environmental SEM^12^, or cryo-techniques^13^,^14^. Critical point drying permitted conductive coating of biological specimens, giving reduced charging as well as improvements in contrast, but the specimens suffered from cracking artifacts and shrinkage of up to 50%, while freeze-drying frequently causes distortion and damage due to ice crystal formation^15^. Flash freezing or high pressure freezing is often used to reduce ice crystal formation in biological specimens. Prior to SEM observation these frozen hydrated samples can either be cryo-sectioned, or mounted whole on a cryo-stage: in which case ion beam milling can also be used to investigate interior structure^13^,^14^. Cryo-TEM, a more advanced variant of this technique, can also be used to investigate frozen-vitrified samples in the TEM. This requires that specimens be maintained at temperatures below ~ −150 °C to remain in an amorphous state and avoid ice crystal damage. Cryo-TEM is ideal for the investigation of macromolecular structures including viruses, however cryo-TEM gives a relatively small field of view, requires a high concentration of virus (~1 mg/ml) and most bacteria are too thick for high resolution TEM imaging in a layer of ice, which can obscure detail^16^,^17^,^18^. Specialised “wet-SEM” or “wet-TEM” specimen holders have also been developed for imaging of fully hydrated samples, but they require highly specialised equipment, and work in scanning-transmission (STEM) mode, so are not directly comparable with SEM. These holders have a liquid chamber or fluid cell isolated from the vacuum by one or two electron-transparent windows: the need for the electron beam to pass through a solid window will always compromise resolution as compared to SEM, where the specimen surface is illuminated directly^9^,^10^,^11^. Another approach for imaging wet samples is known as environmental SEM (ESEM), where differential pumping allows the pressure around the sample to be increased to 10–20 torr. Bacteria have been observed by ESEM, but features such as flagellae are not well resolved, and drying still occurs at these higher pressures, so that ESEM is generally more useful for larger specimens observed below 1000 x magnification^12^. The above mentioned techniques, while giving excellent results with the appropriate specimen, are less suited for microbial investigations, including detection and characterisation, where optimum resolution and a large field of view is required. They also require more complex, specialised and expensive equipment, such as microscopes with field emission illumination sources and/or cryogenic equipment, as compared to a standard SEM instrument. The method presented here is relatively simple, quick to perform (~15 minutes), and can be used with any SEM including those with a standard thermionic tungsten filament. It can be performed on unfixed and fully hydrated specimens. Filtration also allows investigations of specimens that have a low initial concentration of particles^7^.

To produce an electrically conductive surface for SEM, biological specimens are often coated using thin film evaporation or sputtering of carbon or metal in a vacuum coater, which requires prior dehydration of the specimen. This coating process can obscure fine ultrastructural details, depending on the thickness of the layer deposited (usually 2–20 nm). These conventional procedures are difficult to carry out on typical microbiological specimens, which are usually suspensions of small biological particles in water (<100 nm for most viruses, or in the sub-micrometre size range for many bacteria, fungi, and parasites). An additional problem is that the microbes of interest in patient specimens or environmental samples may be present in relatively low concentrations, making observation of them on a surface difficult.

In this report we describe methods for concentrating microbial suspensions for SEM observation on pre-coated filter substrates. We show that, instead of sputter coating, an ionic liquid (1-butyl-3-methylimidazolium tetrafluoroborate) diluted in water can be used to rapidly infiltrate a microbiological SEM sample, forming an electron-lucent conductive surface, which prevents specimen charging and gives good results with microbial specimens (Fig. 1). Ionic liquids are highly conductive salts that remain in the liquid state at room temperature and have a negligible vapour pressure (≤5 × 10^−9^ Torr). Under the high vacuum conditions of a modern SEM (≤1 × 10^−6^ Torr) ionic liquids remain in a liquid state, and do not evaporate during operation, while still being conductive^19^,^20^,^21^,^22^,^23^. The most useful ionic liquids for applications in biological SEM have an electrical conductivity of around 100 mScm^−1^, are electrochemically stable (having an electrochemical window of around 5.8 V), as well as being water soluble and are easily synthesised^24^. Ionic liquids with these properties have been previously demonstrated to give SEM image contrast comparable to the use of metal and carbon coating when used with insulating specimens^19^,^25^. They have also been used for macroscopic imaging of biological specimens, such as seaweed, tissue cultured cells, and condensed chromosomes^20^,^21^,^22^. Conductive substrates such as indium-tin oxide, aluminum foil, or metal coated coverslips have been used to prevent charging^20^, however, these materials are unsuitable for filtration for SEM investigations of microbes. We discovered that, for optimum results using ionic liquid with subcellular objects such as viruses or bacterial flagellae, prior coating of polycarbonate filters with aluminium or gold was necessary. We did not detect any specimen drift when using ionic liquid stained biological specimens, since they were well supported by the conducting membrane used during the initial filtration process. The SPI-pore polycarbonate filters are hydrophilic, and remain so after metal coating, making them an ideal substrate to work with hydrated biological samples. Ionic liquid staining can also be performed within a biological safety cabinet, providing a rapid and safe alternative to sputter coating when working with infectious samples, since vacuum coating equipment can cause aerosols and is not easily contained^20^,^21^,^22^. We have elegantly solved the problem of concentrating the sample, and preventing charging, by metal coating of the filter substrate itself prior to applying the biological sample (Fig. 2). In the absence of thin film coating of the samples, infiltration with ionic liquids was also required to avoid charging. The results are comparable with the use of SEM with sputter coating and TEM using negative staining technique (Figs 1 and 2: Supplementary Figs S1–S5). Ultra-filtration is an important step since it helps to remove debris that can obscure the details of viruses or bacteria present in biological samples. In the current report, we demonstrate clear imaging of viruses and bacterial flagellae in uncoated SEM specimens, which previously required dehydration and sputter coating to achieve, thus extending the resolution and range of microbial samples that can be investigated with SEM.

The images of bacteria stained with ionic liquid had a smoother surface topography than those that were dehydrated and sputter coated. Size measurements show that the dehydrated specimens shrank by approximately 10–20 per cent (Table 1). We interpret the surface detail on the dehydrated, sputter coated bacteria as wrinkling of the cell wall due to shrinkage, rather than observation of additional features that are present *in vivo*: these wrinkles are thus likely to be artefacts due to drying. Bacterial flagellae were also clearly visible with ionic liquid treatment on the conducting substrates (Fig. 1, Supplementary Fig. S3). These results were comparable to those we observed with SEM-sputter coating, and TEM-negative staining.

Ionic liquid techniques can also be used safely with infectious pathogens, in a biologically contained SEM enclosure, allowing the characterisation of novel infectious agents in a condition closer to their hydrated “native state” than by conventional sample preparation techniques^8^. In the case of this investigation our conventional protocol involved dehydration in ethanol series, followed by air drying and metal coating before SEM imaging. For the ionic liquid protocol, the biological sample had a drop of a 2.5% aqueous solution of 1-butyl-3-methylimidazolium tetrafluoroborate placed directly on it. After blotting to remove excess fluid the wet sample was then placed directly in the SEM. When dealing with infectious samples, an additional aldehyde fixation step is needed for the conventional procedure to avoid the risk of infectious aerosols that could be generated during the sputter coating process. This fixation step is not required with the ionic liquid technique, since the sample can be processed in a biological containment hood, and then be placed directly in a SEM in a biologically contained SEM enclosure^8^, for imaging in an unfixed, hydrated state, which is much closer to the native state of the organism. Microscopy complements conventional diagnostic tests that can miss novel or variant strains^3^,^26^,^27^,^28^ and can rapidly identify the type of organism present, guiding the selection of more specific tests^29^. However, electron microscopy usually requires a minimum concentration of particles for reliable identification of microbes. For viruses, this is between 10^5^ to 10^6^ virus particles/mL^4^,^5^. With filtration techniques, both TEM and SEM of viruses can be carried out with as little as 5000 particles per sample^7^.

Ionic liquid staining on pre-coated filters is widely applicable to any biological sample which can benefit from filtration to concentrate particles of interest. The use of metal coated filters with more than one type of coating on different areas allows selection of whichever of these coatings gives the optimum results for observing a particular specimen or specific features, and helps to save time (Fig. 2a,e–k). For example, after ionic liquid staining bacterial flagellae appeared brighter against the Al-coated substrate, while on the Au-coated area of the filter the contrast is reversed and the flagellae appeared darker (Fig. 2h–k). Similarly the images of Ebola virus and *Leptospira biflexa* showed good quality topographic detail when imaged with an aluminum coated filter, but the biological material had less detail and appeared as a dark flat silhouette when imaged on gold coated filters (Supplementary Figs S4 and S5). We propose that this is due to the higher secondary electron emission signal from Au as compared to Al. In this investigation we collected SEM images with the secondary electron detector, which is most commonly used for routine imaging with biological specimens. In SEM, the secondary electron emission coefficient (δ) is relatively constant regardless of atomic number. However an exception is with Au, for which δ is almost twice that of Al and many other elements. The value of δ is also affected by beam energy: at 20 kV, δ is 0.1 for Al, and 0.2 for Au^30^. By measuring the intensity in secondary electron images taken at 4 kV with both Al and Au in the same image (Fig. 2f), we calculated the signal from the Au to be 2.1 times the intensity of the Al, which is close to that expected theoretically. With the images recorded of specimens on the gold coated filters, we interpret the results as producing too much contrast from the background substrate, which tended to obscure fine details such as flagellae which appear as a “silhouettes” on a bright background. However, the ionic liquid infiltrated microbes, and the aluminum coated filter, have similar emission coefficients, thus the contrast is largely due to the topography rather than the differences in material composition, allowing more fine details to be seen.

Pre-coating of the filters with metal did not affect the pore sizes, or the filtration capacity of the filters (Fig. 2). The entire ionic liquid staining protocol can be carried out on a standard laboratory bench in about 15 minutes, and fits inside a biosafety cabinet (Fig. 2c,d). In sputter coating for SEM, too thin a layer causes poor conduction and charging, while too thick a layer obscures fine details. The thickness used for pre-coating the substrates can be much greater than sputter coatings typically used for biological specimens, to ensure good conductivity, so long as the filter pores are not blocked. (Fig. 2e–k, Supplementary Fig. S2).

In this investigation we used coatings of 18 and 27 nm for Al and Au respectively, since these thicknesses were found to be sufficient to prevent charging, as compared to uncoated filters (Supplementary Fig. S2). Substrates with these minimum thicknesses were easily selected since they were visible as a shiny metallic coating. When coatings of less than 27 nm for Au or 18 nm for the Al were present, they had an opaque or flat-white appearance (Fig. 2). With ionic liquid staining using these metal coated filters, we were able to visualize the fine structural details of bacteria, such as the flagellae of *Salmonella*, which are 20 nm in diameter, by SEM (Fig. 2j,k, Supplementary Fig. S3).

The results obtained with filtration and simple ionic liquid infiltration for SEM are very comparable in quality with those from conventional sputter coating in SEM, and negative staining in TEM for a variety of bacterial and viral specimens, including *Leptospira*, *Salmonella*, vaccinia virus, and Ebola virus (Fig. 1, Supplementary Fig. S3–S5)^7^,^16^,^31^,^32^. We found that there was much less shrinkage of the ionic liquid infiltrated bacteria and viruses as compared to both the dehydrated sputter coated SEM preparations, and the TEM negative stained images (Table 1). In all cases the dimensions of the dehydrated SEM sputter-coated and negative-stained TEM microbes were from 9.9% to 18.9% smaller than the ionic liquid treated specimens (Table 1). In a previous investigation we imaged frozen-vitrified Ebola virus by cryo-electron microscopy the diameter of the Ebola virus was measured as 96–98 nm^16^ which is very similar to the value of 98.5 ± 10.2 nm for the diameter measured of the same specimens treated with ionic liquid in the present study. This further demonstrates that the volumes of the ionic liquid infiltrated samples are comparable to those measured under frozen hydrated conditions, and closely reflect the fully hydrated native state of Ebola virus. For a bacilliform structure, a 10 per cent reduction in dimensions is equivalent to a 27% reduction in volume due to dehydration, though collapse and flattening of the cylindrical shape would imply an even greater degree of water loss. It is clear from that this flattening and collapse due to dehydration is present to some extent in all of the images of sputter coated viruses and bacteria (Fig. 1).

Although the images appear relatively similar, gold sputter coating appears to give slightly more contrast than ionic liquid. Another observable difference is less of surface roughness of the bacterial cell walls in the ionic liquid stained images. This can be seen in the images of *Salmonella* (Fig. 1, Supplementary Fig. S3). In these images there is a clear textured and wrinkled appearance on the surface of the sputter coated bacterial cells, and a smooth appearance on the cell walls of the ionic liquid infiltrated preparations. We propose that this observed difference is largely the result of loss of cell turgor due to dehydration and volume loss in the sputter-coated specimens, and thus the wrinkles may actually be an artifact or feature that is accentuated by dehydration. Supporting evidence for this comes from the fact that other fine structures, such as flagellae, are clearly visible (and of similar appearance) in both the sputter-coated and ionic liquid treated specimens. Thus, the results of previous studies using sputter coating of bacteria may have to be cautiously re-interpreted in the light of possible dehydration effects.

The ionic liquid procedure presented in this investigation is rapid and reproducible since specimen filters can be prepared in advance. As the ionic liquid has a very low vapour pressure, an added benefit is that drying artifacts such as shrinkage, wrinkling or cracking that can occur during SEM observation are avoided (Table 1, Supplementary Fig. S3). In the future, we anticipate the development of a variety of different types of filter coatings to further improve SEM techniques using ionic liquid staining for biological specimens in the nanometre size range.

## Methods

### Bacterial growth

*Salmonella* Senftenberg (kindly provided by Dr. George Golding, National Microbiology Laboratory) strains were grown overnight in 3 mL LB broth at 37 °C with shaking. Fifty μL of bacteria were then sub cultured into 3 mL LB broth and grown at 37 °C with shaking for 4 hours to the approximate mid-log phase of growth. The bacteria were fixed at 1:1 v/v in 1% paraformaldehyde and 2% gluteraldehyde for 1 hour at room temperature. *Leptospira biflexa* serovar Patoc (kindly provided by Dr. Robbin Lindsay, National Microbiology Laboratory) was grown in Ellinghausen and McCullough media modified by Johnson and Harris (EMJH) (Royal Tropical Institute, The Netherlands). The inoculate culture was placed at 30 °C for 14 days and then stored at room temperature in low light.

### Growth and Purification of Modified vaccinia Ankara Virus

Baby hamster kidney fibroblast cells (BHK-21:ATCC) were grown to 80% confluence in high-glucose Dulbecco’s Modified Eagle’s Medium supplemented with 10% fetal bovine serum and 1X Penicillin-Streptomycin. The BHK-21 cells were infected with 1 mL of Modified vaccinia Ankara (MVA) virus (kindly provided by Dr. Jingxin Cao, National Microbiology Laboratory) and incubated for 48 hours at 37 °C with 5% CO_2_. The infected cells underwent 3 freeze-thaw cycles in the presence of the growth media, alternating between −80 °C and room temperature. MVA was collected in the supernatant after removing cells and debris by centrifugation at 3000 × g for 3 min.

### Growth and Purification of Ebola virus

Zaire Ebola virus (kindly provided by Dr. Steven Jones) was propagated, purified and rendered non-infectious as previously described^16^,^33^.

### Gold Coated Sample Preparation for SEM Imaging

Unless otherwise stated sample preparation was performed in a class II biosafety cabinet. All samples were passed through SPI-pore polycarbonate track etch filters (SPI Supplies, West Chester, PA, USA) held inside a 13 mm Swinnex® filter unit (Millipore, Billerica, MA, USA). For bacterial sample preparation, 0.08 μm pore size filters were used, while the 0.05 μm pore size was used for MVA and Ebola virus preparations. Filters were first wetted using 2 mL Dulbecco’s phosphate buffered saline (DPBS) in a 3 mL Luer-Lok™ syringe. To load the sample onto the filter, 100 μL of sample was added to 5 mL DPBS in a 5 mL syringe. After attaching the syringe to the filter holder, the sample was filtered through the filter using a Legato 200 syringe pump (KD Scientific, Holliston, MA, USA) at a rate of 1000 μL/min. In some cases the concentration of the sample was too high which would overload the filter and fluid could not flow through. If such an event occurred the sample was diluted 1:5 or 1:10 until fluid could flow freely through the filter. The sample filters were washed using 3 mL syringes containing 2 mL each of 50%, 70%, 85%, 95% and 100% ethanol in increasing concentration. Following the last wash the filter was removed from the filter unit and allowed to air dry for 30 min. The filter was trimmed, cut in half and placed on a 9 mm carbon disc (SPI Supplies) and mounted on a 3/8” aluminum stub (SPI Supplies). Flash Dry silver paint (SPI Supplies) was used on each of the four corners of the filter to create a contact between the filter and the stub. The samples were removed from the biosafety cabinet and sputtered with gold using a Quorum Q150R S (Quorum Technologies, East Sussex, UK) containing a 0.1 mm gold target. The sample was pumped down, purged with argon and sputtered with gold for 120 sec on a rotating stage.

### Preparation of Metal Coated Polycarbonate Filters

For a filter with a single type of metal coating the following procedure was used (Supplementary Fig. S1a). For the forts step either 2 cm of 0.2 mm diameter Al wire or 3 cm of 0.2 mm Au wire was wrapped around the tungsten filament. SPI-pore polycarbonate track etch filters were placed on a filter paper in a glass dish and secured from movement with metal washers. The dish was then placed in a Turbo Carbon Coater (Agar Scientific Ltd, Stansted, Essex, United Kingdom). Once under vacuum, the voltage was gradually increased until the Al or Au wire had completely evaporated. The 2 cm of aluminum wire would create a metal film approximately 18 nm thick, and the 3 cm of Au would create a metal film approximately 27 nm thick. To produce a filter with two types of metal on it the following variant of the above procedure was used (Supplementary Fig. S1b). The filter was placed on top of the filter paper on the glass dish, and a razor blade was placed directly over one half of the filter. One of the above metals (Au or Al) was evaporated as described above. Following this, the razor blade as removed and a second razor blade was used to cover the region of the filter where the previous metal was evaporated, and the remaining area of the filter was then coated with the second metal.

### Ionic Liquid Sample Preparation for SEM

The ionic liquid 1-butyl-3-methylimidazolium tetrafluoroborate was diluted to a final concentration of 2.5% in distilled water and placed at 40 °C for 30 min to reduce viscosity^9^. The metal-coated filters were placed inside the filter unit and wetted using 2 mL DPBS. A total of 100 μL of sample was added to 5 mL DPBS and filtered through the coated filter using the Legato filter pump. After the sample was washed with 5 mL distilled water the filter was removed from the filter assembly and excess liquid was wicked off with tissue paper. The filter was immediately trimmed, placed on a carbon disc mounted on an aluminum stub. The four corners of the filter were painted to the aluminum stub using Flash Dry silver paint. A total of 50 μL of ionic liquid was pipetted onto the sample and the excess was removed after 60 sec by blotting with filter paper. The samples were then viewed by SEM.

### SEM Sample Imaging

All samples were imaged in a JCM-5700 Scanning Electron Microscope (JEOL USA, Peabody, MA, USA) contained inside a mobile biological containment enclosure (Dycor Technologies Ltd, Edmonton, AB, Canada)^8^. Gold coated specimens were imaged under high vacuum at 6 kV, with an 8 mm working distance and a 30 μm objective lens aperture. Images were collected using the secondary electron detector, the acquisition time per image was 160 sec and each image was 2560 × 1920 pixels. Images of ionic liquid stained samples were obtained using the above noted settings with the exception that the acceleration voltage was adjusted to 4 kV. SEM images were recorded at magnifications ranging from 3,000 x to 20,000x.

### TEM Sample Preparation and Imaging

Samples were adsorbed for 1 min to a formvar film on a carbon-coated 400-mesh copper grid. The adsorbed samples were washed 3X in distilled water and negatively contrasted with 2% methylamine tungstate (Nano-W; Nanoprobes, Yaphank, NY, USA). Imaging was performed at 200 kV using a FEI Tecnai 20 transmission electron microscope (FEI Company, Hillsboro, OR, USA). Digital images of the specimens were acquired using an AMT Advantage XR 12 CCD camera (AMT, Danvers, MA, USA). TEM images were recorded at magnifications of 3,500 x to 19,000x.

### Image processing: length measurements

Ebola virus, *Salmonella* Senftenberg, and *Leptospira biflexa* diameter measurements were made using the Image J software package^34^ using the straight line tool, and the analyze/measure function. Length measurements were calibrated using the scale bars on the image, and the analyze/set scale function in Image J. For this analysis, diameter measurements only were made, since the bacteria and viruses have varying lengths, but relatively constant diameters. Measurements were collated and analysed using MS Excel.

## Additional Information

**How to cite this article**: Golding, C. G. *et al.* The scanning electron microscope in microbiology and diagnosis of infectious disease. *Sci. Rep.* **6**, 26516; doi: 10.1038/srep26516 (2016).

## Supplementary Material

Supplementary Information

## Figures and Tables

**Figure 1:**
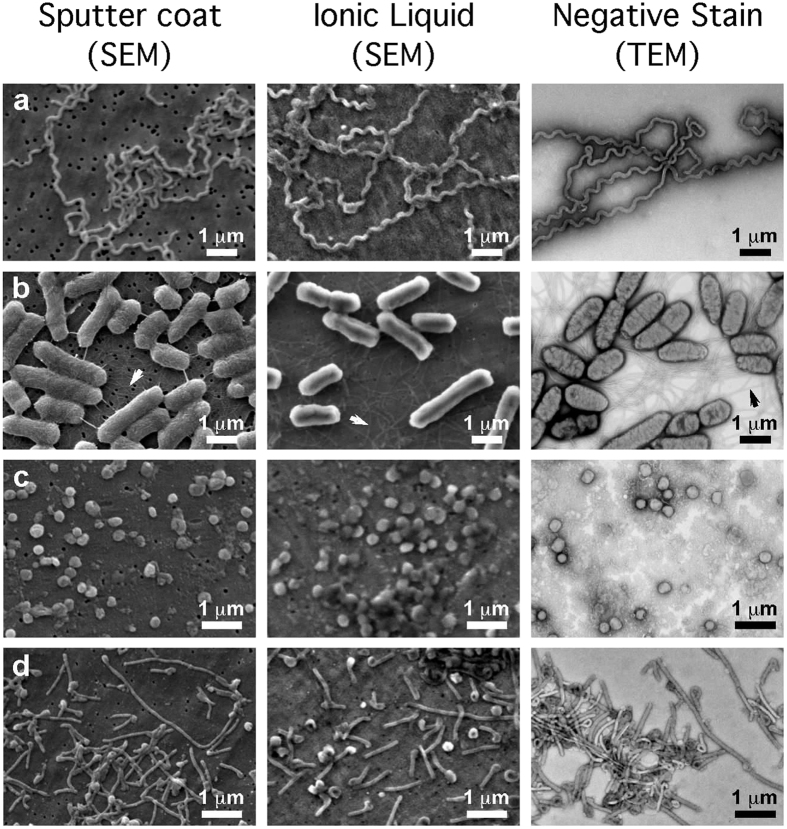
Comparison of conventional sputter coating SEM sample preparation methods (panels on the left-hand side) with ionic liquid treatment (centre panels) and conventional TEM (panels on the right-hand side) for the observation of microbes: (**a**) *Leptospira biflexa*, (**b**) *Salmonella* Senftenberg, (**c**) vaccinia, and (**d**) Ebola virus. The SEM images in the left-hand side panels were of specimens that were sputter coated with gold, on plain uncoated filters. Images in the centre panels were of specimens treated with ionic liquid after deposition on pre-coated aluminum filters. On the right-hand side, TEM images of similar specimens prepared using methylamine tungstate negative staining.

**Figure 2:**
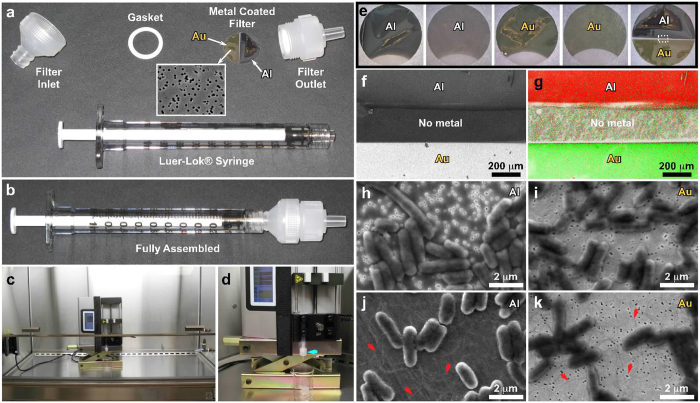
Preparation of biological samples for SEM. (**a**) The components of the filter unit are shown before assembly. The inset SEM image shows a gold coated filter at high magnification before a specimen is applied. Note that the filter is clean and the pores are clearly evident. (**b**) The filter unit is shown after assembly, and in use with a syringe pump in a biosafety cabinet (**c,d**). The blue arrow in (**d**) points to the filter unit. (**e**) Images of filters that have had metal evaporated on them. The thickness of the Al is 18 nm, and 9 nm, and the Au is 27 nm and 9 nm thick. For the filter with both metals the thickness of the Al and Au are 18 nm and 27 nm, respectively. (**f**) SEM and (**g**) the corresponding elemental map generated by X-ray microanalysis of a region similar to the one highlighted by the dotted rectangle in (**e**). (**h–k**) SEM images of *Salmonella* stained with ionic liquid illustrating the effect of different metal types, and thickness of metal evaporated on the final images recorded (**h** Al 9 nm, **i** Au 9 nm, **j** Al 18 nm, **k** Au 27 nm). Red arrows indicate flagellae.

**Table 1:** Width measurements of microbes.

Specimen	Sample preparation	Electron Imaging	Width (nm)	N	Relative Size
*Salmonella* Senftenberg	Ionic liquid	SEM	627.1 ± 50.9	193	–
*Salmonella* Senftenberg	Sputter coating	SEM	564.9 ± 44.4	185	90.1%
*Salmonella* Senftenberg	Negative Stain	TEM	563.6 ± 43.5	174	89.9%
*Leptospira biflexa*	Ionic liquid	SEM	156.2 ± 15.8	153	–
*Leptospira biflexa*	Sputter coating	SEM	129.7 ± 14.1	158	83.0%
*Leptospira biflexa*	Negative Stain	TEM	126.8 ± 17.0	164	81.8%
Ebola virus	Ionic liquid	SEM	98.5 ± 10.2	123	–
Ebola virus	Sputter coating	SEM	85.6 ± 8.4	141	86.9%
Ebola virus	Negative Stain	TEM	82.8 ± 5.0	146	84.1%

The effect of sample preparation methods on microorganism size. N = number of measurements.
