# Supplementary material for: The scanning electron microscope in microbiology and diagnosis of infectious disease
Source: Sci Rep. 2016 May 23;6:26516. doi: 10.1038/srep26516 (PMC4876401; doi:10.1038/srep26516)
Supplement: Supplementary Information [file srep26516-s1.pdf]

Supplemental Information

The scanning electron microscope in microbiology and diagnosis of infectious disease

Christine G. Golding[1], Lindsey L. Lamboo[1,2], Daniel R. Beniac[1,*], and Timothy F. Booth[1,2,*]

1 Viral Diseases Division, National Microbiology Laboratory, Public Health Agency of Canada,Winnipeg, Manitoba, Canada.
2 Department of Medical Microbiology, University of Manitoba,Winnipeg, Manitoba, Canada.

*** E-Mail: tim.booth@phac-aspc.gc.ca; daniel.beniac@phac-aspc.gc.ca**

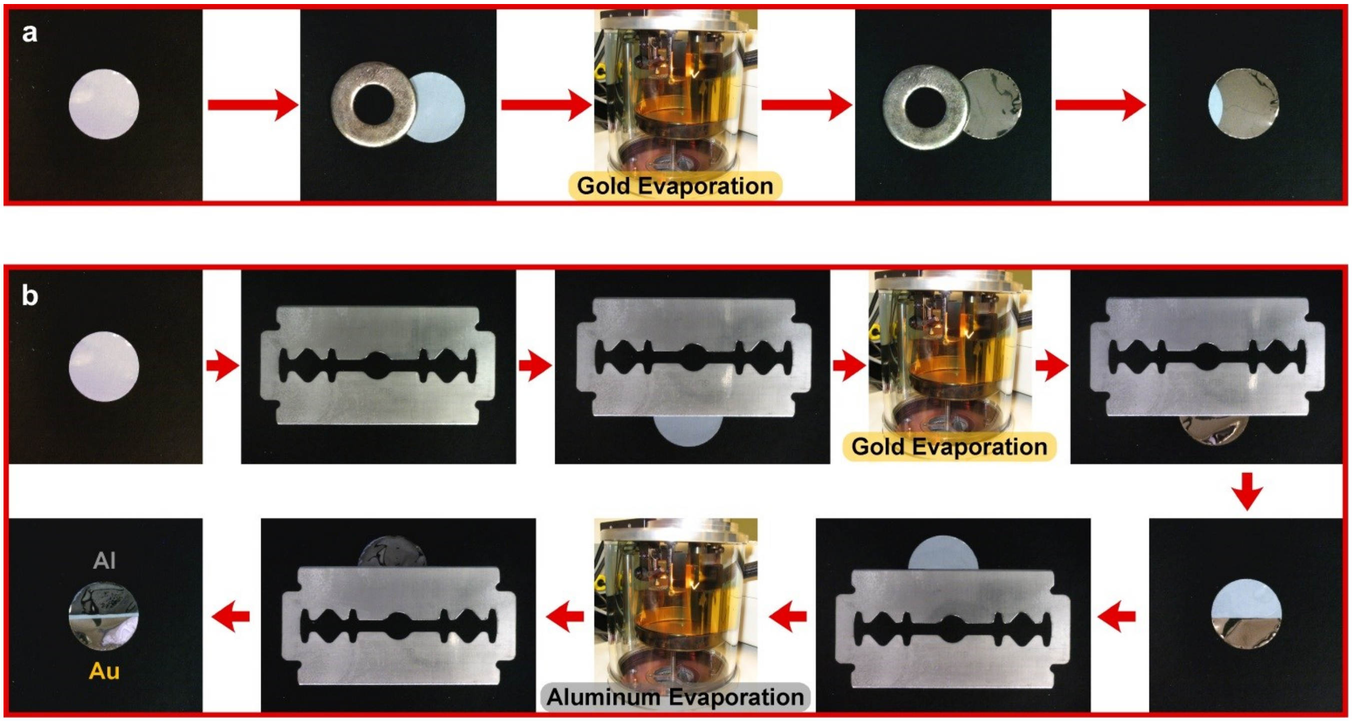

Supplemental Figure S1. Fabrication of single and dual metal coated SEM filters. SPI-pore polycarbonate track etch filters require a conductive metal coating for SEM imaging. This process does not affect the ability of the membrane filter to filter fluid samples. (**a**) To produce a filter with a single metal coating, a washer is placed on the edge of the filter to hold it in place in the evaporator. After metal evaporation a filter is produced with the majority of it covered with metal. The metal coated region is used for SEM imaging. (**b**) To produce a dual coated filter, half of the filter is covered by a razor blade and the other half of the membrane is subsequently coated with metal. This process is then reversed and the other half is coated by a different metal. This produces a dual coated filter membrane. In this example a filter was produced with gold on one side and aluminum on the other side.

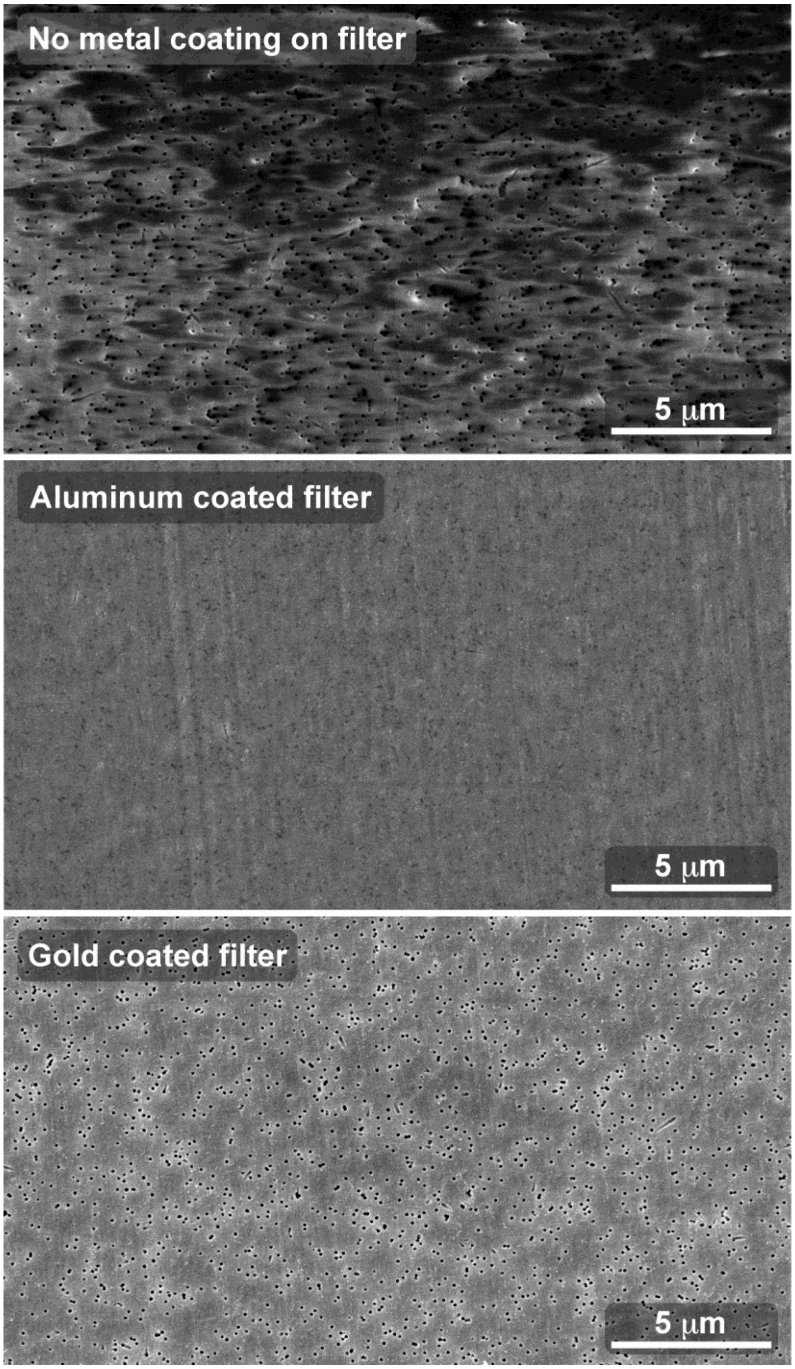

Supplemental Figure S2. The effect of metal evaporation on SEM filters. Three SEM images were recorded with the same imaging conditions. The only difference was if the filter had metal evaporated on it. The uncoated filter exhibited charging effects, whereas the gold and aluminum coatings resulted in high quality images. Furthermore, the evaporation of metal onto the filter had no impact on the membranes ability to filter fluids. This indicated that the metal evaporation procedure did not plug the pores in the filter.

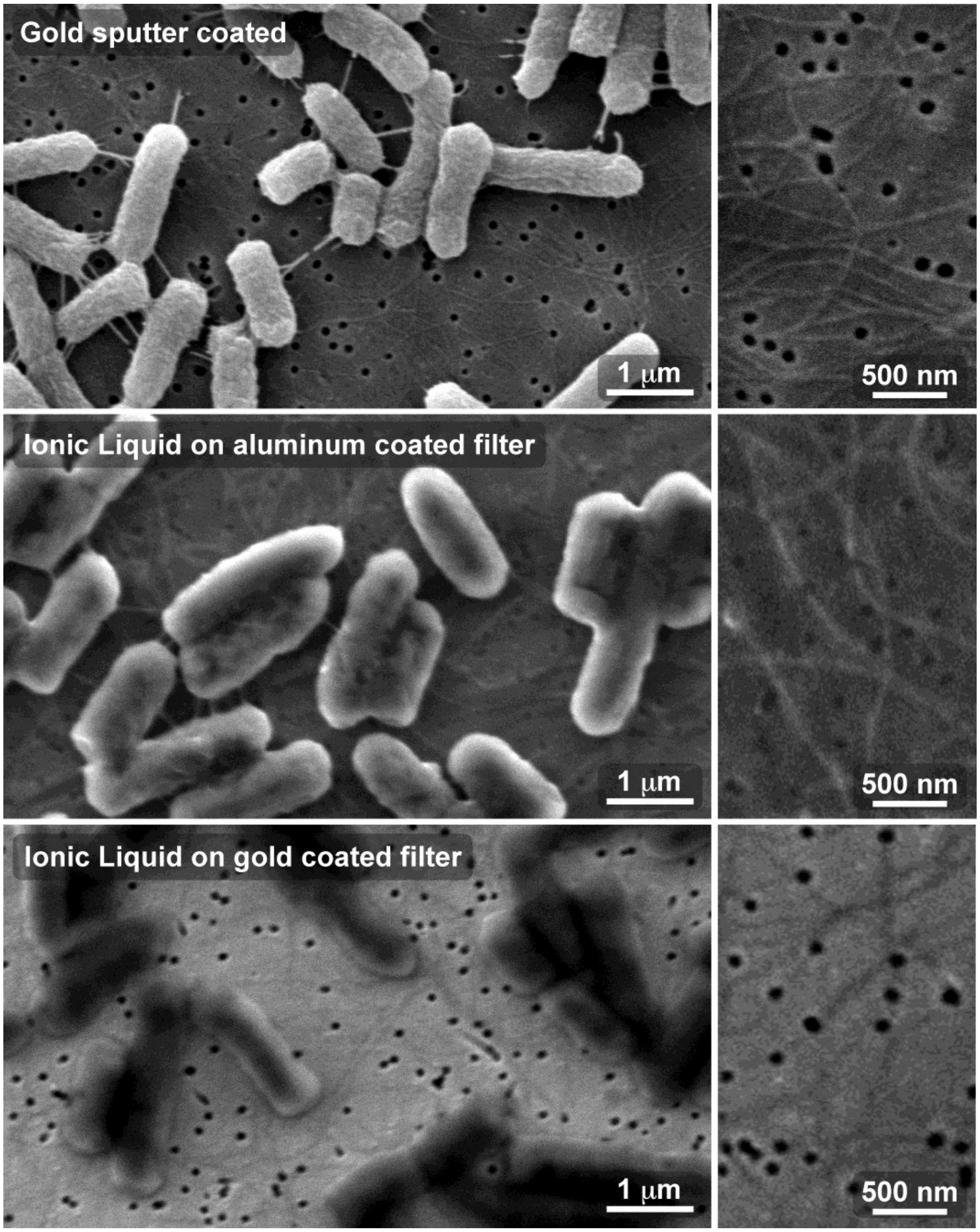

Supplemental Figure S3. SEM images of *Salmonella* (non-Typhi). Six images are presented to compare samples prepared by the established technique of sputter coating, versus the use of ionic liquid staining on both aluminum and gold coated filters. The sputter coated samples go through an alcohol drying procedure to eliminate water. Whereas, the ionic liquid treated samples are stained and imaged in a hydrated state. The aluminum and gold coated samples generate similar results, with a reversal of contrast for the flagella on the metal coated filter. High magnification images of the flagella are presented on the right.

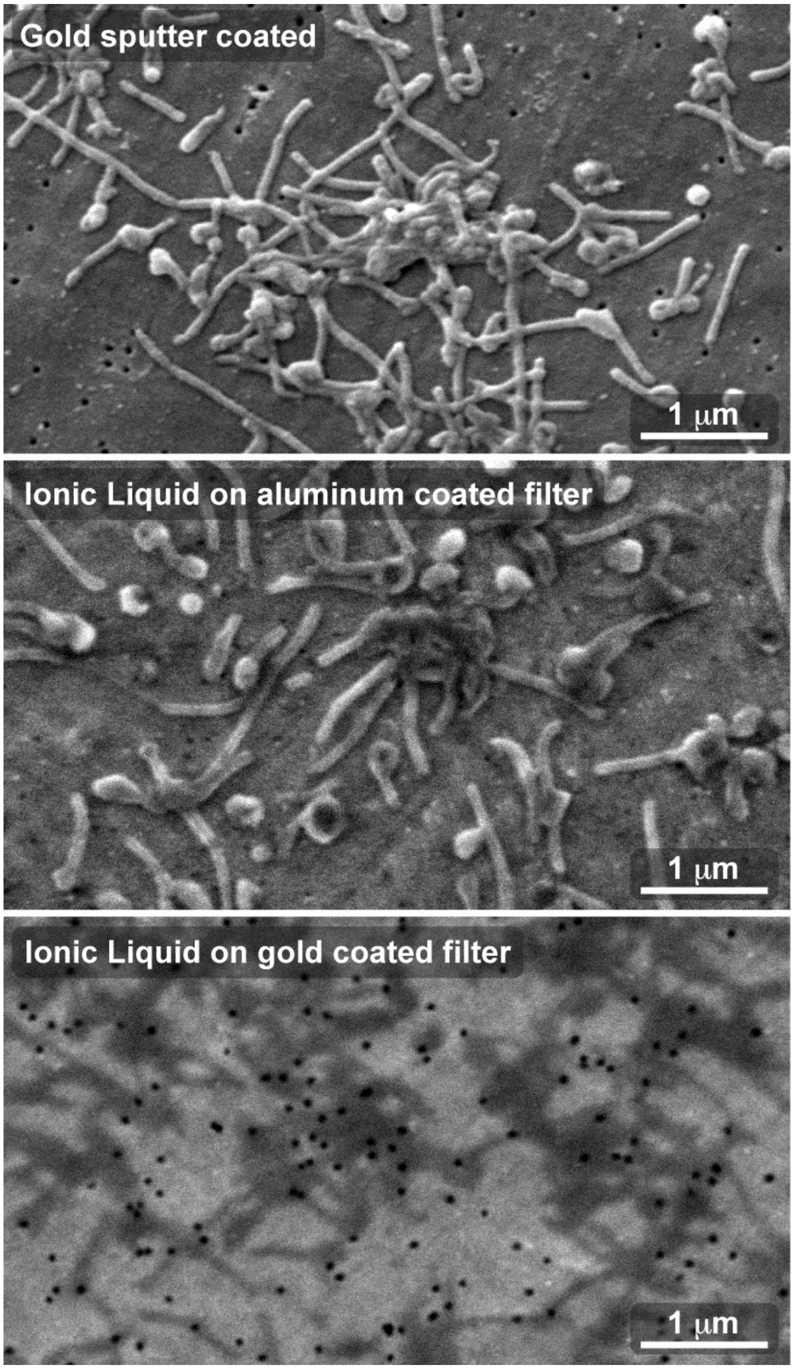

Supplemental Figure S4. SEM images of Ebola virus. Three images are presented with the exact same combination of conditions as presented in Supplemental Fig. S3. In this case there is a dramatic difference between the ionic liquid results for the gold and aluminum coated filters. The aluminum coated filter generates a similar three dimensional appearance for the Ebola virus as was seen for the sputter coated sample. The Ebola virus appeared as a dark silhouette when the gold coated filter was used.

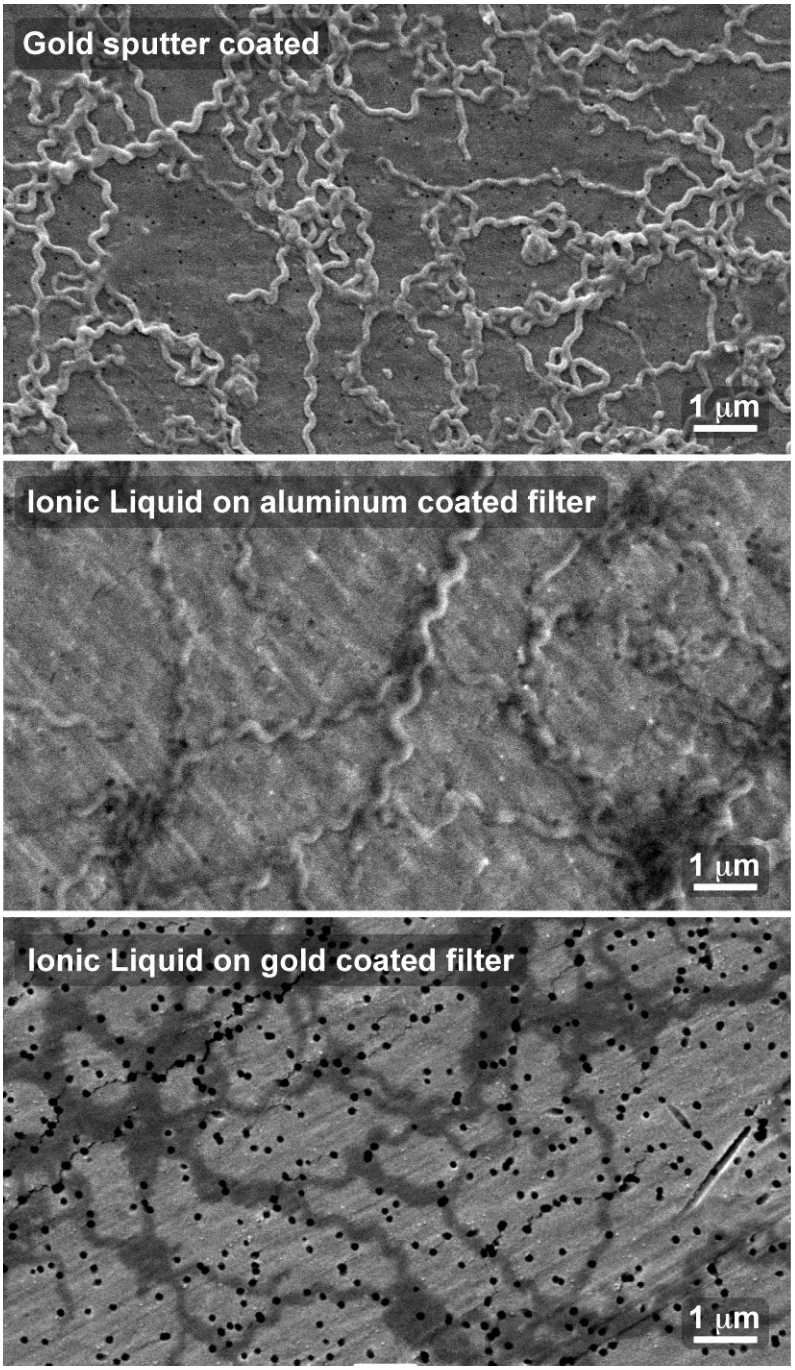

Supplemental Figure S5. SEM images of *Leptospira biflexa*. Three images are presented with the exact same combination of conditions as presented in Supplemental Fig. S3. The results generated are comparable to those seen for the Ebola virus in Supplemental Fig. S4. In both cases the aluminum coated filters generated images with three dimensional topography. The gold coated filter generated images with a dark flat silhouette outline of *Leptospira biflexa*, which was similar to the images generated for the Ebola virus.
